# Sensory Processing in Williams Syndrome: Individual differences and changes over time

**DOI:** 10.1007/s10803-021-05197-0

**Published:** 2021-07-21

**Authors:** Bradley Powell, Jo Van Herwegen

**Affiliations:** Department of Psychology and Human Development, UCL Institute of Education, 20 Bedford Way, London, WC1H 0AL UK

**Keywords:** Williams syndrome, Sensory processing, Sensory registration, Hyperacusis, Longitudinal

## Abstract

This study examined individual differences as well as the development of sensory processing difficulties in children with Williams syndrome (WS) using a cross-sectional (Experiment 1) and longitudinal design (Experiment 2). In Experiment 1, a clustering approach of sensory processing scores suggested two groups. Experiment 2 showed that the clusters identified in Experiment 1 were not stable across development, especially for those with high sensory impairments at either time point. Yet, most children experienced high impairments in sensory registration at both time points, suggesting impaired registration is a core phenotype of sensory processing in children with WS across development. Possible mechanisms, limitations and implications are discussed.

## Introduction

Williams Syndrome (WS) is a rare neurodevelopmental disorder, caused by a genetic deletion on the long arm of chromosome 7 with a prevalence of around 1 in 20,000 live births (Martens et al., 2008). The cognitive profile of individuals with WS is typically uneven, with better language and face recognition skills in contrast to poorer planning and visuo-spatial abilities. In addition, individuals with WS tend to have mild to moderate intellectual difficulties with an average IQ of 55, although, there is much variability in cognitive ability (Miezah et al., 2020). Behaviourally, WS is characterised by hyper-sociability; an intense drive to form bonds with others and little fear of strangers (Shore et al., 2017). However, individuals experience social difficulties, such as problems maintaining friendships and understanding others’ intentions (Fisher & Morin, 2017). It is also widely documented that individuals with WS experience hyperacusis or increased sensitivity to certain sounds. Yet, it remains less clear to what extent individuals with WS experience general sensory processing abnormalities as a systematic review has shown that only four studies thus far have examined sensory processing in WS more broadly and none have examined changes over development (Glod et al., 2019). Given that such abnormalities can be detrimental to long-term development (Carvill, 2008; Dellapiazza et al., 2020) and can contribute to increased anxiety (Uljarević et al., 2018) and behavioural difficulties (Wuang & Tsai, 2017), additional research is needed to understand the prevalence of general sensory processing difficulties within WS over development.

## Sensory Processing

Sensory processing can be defined as the ability to process and manage incoming sensory information (e.g. visual, auditory, vestibular, proprioceptive) in the cerebral cortex and brainstem to facilitate adaptive and congruous responses within the environment regarding to this information (Baker et al., 2008). The definition encapsulates the process of distinguishing input from the senses as well as sensory modulation, which includes the ability to regulate our arousal from sensory information (Dunn, 1997; Nakagawa et al., 2016). Dunn (1999) proposed a Sensory Processing Framework which provides a model to categorise an individual’s responses to sensory information based upon their neurological threshold to sensory input and their method of self-regulation. The framework consists of four sensory quadrants, which are: (1) registration (high threshold and passive self‐regulation), characterised by not noticing certain sensory input or responding slowly, (2) seeking (high threshold and active self‐regulation), characterised by the desire to experience more sensory stimulation, (3) sensitivity (low threshold and passive self‐regulation), characterised by distractibility and discomfort by sensory stimuli and (4) avoiding (low threshold and active self‐regulation), characterised by acting to reduce or prevent exposure to stimuli (Dunn, 1999). Difficulties can occur across multiple sensory domains and can be influenced by the individuals emotional state (Crane et al., 2009). These difficulties are often measured using the Sensory Profile Questionnaire (SPQ; Dunn, 1999), which provides information on a number of factors: an individual’s capacity to process incoming sensory information, their ability to modulate such information, and the effect sensory input has on their emotional and behavioural state. By utilising items from across each of these scales, four additional scores are provided which represent the four sensory quadrants. Research has documented that for individuals with developmental conditions, including autism, ADHD as well as those with intellectual disabilities, sensory processing difficulties are highly prevalent (Little et al., 2018.; Pavão & Rocha, 2017), and the consequences of such sensory disturbances have been shown to lead to poorer adaptive and more disruptive behaviours (John & Mervis, 2010), thereby impacting educational attainment and socialisation (Pastor-Cerezuela et al., 2020; Wuang & Tsai, 2017).

Within a sample of 788 typically developing (TD) children aged 3–14, sensory processing difficulties were found to be experienced by as many as 11%. Yet, the study illustrated that difficulties with general sensory processing abnormalities tend to decrease with age in TD children (Little et al., 2018). In addition to age, there are also a number of individual and environmental factors that may influence the sensory developmental trajectory, such as parenting, interventions and schooling (Allen & Casey, 2017; Ben-Sasson et al., 2013; Kern et al., 2007; Pfeiffer et al., 2011). In addition, studies have shown that although sensory processing difficulties are common in neurodevelopmental disorders, the type of sensory features may differ for some features and overlap for others and these patterns may change with age (e.g., Little et al., 2018).

## Sensory Processing Difficulties in Williams Syndrome

Research is yet to establish whether WS is characterised by a typical sensory processing pattern. A recent narrative review comprised of 18 studies found that 15 studies thus far have explored hyperacusis in WS and that only four have investigated general sensory processing (Glod et al., 2019). Across these studies, it has been reported that approximately 80% of individuals with WS experience sensory processing impairments. In addition, research by John and Mervis (2010), utilising the Short Sensory Profile (SSP) (Tomchek & Dunn, 2007) with a sample of 78 children aged four to 11 years old, found that more than half of the participants experienced definite impairments and scored well below two standard deviations of the mean for typically developing children in the Auditory Filtering (59%), Low Energy/Weak (64.1%) and Under-Responsive/Seeks Sensations (62.8%) scales. This suggests the areas posing the greatest difficulty for individuals with WS consist of screening out sounds, using muscles to move and noticing sensory events. Difficulties with screening out sounds are expected, given the high prevalence of hyperacusis within WS (Gallo et al., 2008; Gothelf et al., 2006; Klein et al., 1990; Udwin, 2010). As many as 80% of individuals with WS have been argued to have hyperacusis, which can cause aversive reactions to incoming stimuli and disrupt adaptive behaviours (Gallo et al., 2008; Gothelf et al., 2006; Klein et al., 1990; Udwin, 2010). The majority of individuals with WS develop hyperacusis during infancy or early childhood, and the severity of the hyperacusis tends to decrease as the child enters late childhood to adulthood (Gothelf et al., 2006; Udwin, 2010).

It has been suggested that difficulties with executive functioning are a primary cause of the sensory processing impairments (Baranek et al., 2006), in that impaired executive functioning systems could disrupt an individual’s ability to exert top-down cognitive control and orient towards relevant sensory stimuli (Costanzo et al., 2013; Little et al., 2017). Related to this, Wuang and Tsai (2017) used the Sensory Profile (SP) (Dunn, 1997) and found within a sample of 38 children with WS (aged 6–12), 81.6% of the sample experienced sensory processing abnormalities and these difficulties were found to correlate with lower participation in school activities and poorer adaptive behaviours.

## Individual Differences in Williams Syndrome

Individuals with WS tend to show uneven cognitive and behavioural patterns and there is wide individual variability within the WS population (Miezah et al., 2020; Porter & Coltheart, 2005; Van Herwegen et al., 2011) including for sensory profiles. John and Mervis (2010) found that participants’ scores on the SSP subscales clustered into two homogenous subgroups of sensory processing ability: *high impairment* (*n* = 31) and *low impairment* (*n* = 40). The *high impairment* cluster had lower mean scores on each of the subscales, indicating greater impairments. The groups significantly differed on all scales, aside from the Movement Sensitivity scale. The two clusters did not differ on chronological age but those in the *high impairment* group had poorer performance on all aspects of executive functioning, more negative and less effortful control temperaments, as well as poorer adaptive functioning and more problem behaviours.

However, it is currently not clear if these clusters remain stable over time and longitudinal research is required to provide further insight into the developmental trajectory of sensory processing within WS.

## Current Study

As such the current study aimed to fill this gap by first of all examining individual differences in sensory processing difficulties related to sensory processing factors in a cross-sectional sample and the extent to which sensory systems were impaired within children with WS over development using a longitudinal paradigm with a sub-set of the children from the cross-sectional study.

The current study examined the following hypotheses:

1. The majority of the sample will experience sensory impairments within the auditory, vestibular, registration and seeking sensory factors, whereas the visual, touch, oral, sensitivity and avoidance scales will be less impaired (John & Mervis, 2010).
2. Participants scores from the SP factors will cluster into two groups of sensory impairments, and those within the low impairment group will display a complex sensory profile consisting of high and low impairments across modalities (John & Mervis, 2010) across both time points.
3. The intensity of the overall and individual sensory profiles will decrease across development (Baranek et al., 2006), if the developmental trajectory of people with WS follows that of typical populations and those with autism (Ben-Sasson et al., 2009).

A two-experiment design was used for this study. Experiment 1 used a cross-sectional design to explore whether there were specific sensory subtypes and processing patterns in children with WS. Experiment 2 included a longitudinal design with a sub-sample of participants from Experiment 1, to explore whether individual and group sensory processing patterns related to the different sensory processing factors at baseline remained stable or changed across development. Further understanding of individual differences in sensory processing difficulties and whether these remain stable across development allows the creation of interventions to facilitate adaptive behaviours and for children with WS to successfully manage their sensory environment.

## Experiment 1

Previous studies have identified that there is considerable variability within the WS cognitive and behavioural profile. One study by John and Mervis (2010) also found individual differences in the sensory processing profile of individuals with WS. Yet, this study used only the short version of the SP which merges sensory modalities across scales and thus provides limited insights into individual differences in sensory processing. As a result, the current study re-examined sensory processing clusters in WS using a more comprehensive measure of sensory processing, the long form SP (Dunn, 1999).

## Participants

Data for this study was obtained from the WiSDom database, a UK population-based study involving the collection of cognitive and behavioural data for individuals with WS (Van Herwegen et al., 2019). Data from 37 children with WS between ages of 3–14 years old (mean = 5 years and 6 months, SD = 2 years and 8 months) was obtained and over half of the sample were males (*n* = 23; 62% male). Each individual with WS had their diagnosis confirmed either by a genetic test or clinical diagnosis. As the data was derived from a longitudinal dataset, additional participant details were not available. This is caused by the fact that WisDom database was put together retrospectively and thus different adaptive functioning and intellectual measures were used for different participants. This project received ethical approval from the Faculty Ethics Committee (REC1323).

## Materials

The SP (Dunn, 1999) is a 125-item parent-report that measures children’s responses to everyday events in six sensory modalities (i.e. auditory, visual, vestibular, touch, multisensory and oral) related to five modulation areas (i.e. endurance/tone, body position and movement, activity levels, emotional responses and visual input affecting emotional responses and activity level) that can be arranged in three emotional and behavioural categories (i.e. emotional/social responses, behavioural outcomes, thresholds for response). The parents responded to each behavioural statement using a 5-point Likert scale, whereby 1 = always, when presented with the opportunity, the child responds in the manner described every time, and 5 = never, when presented with the opportunity, the child never responds in this fashion. The SP also provides scores in the four quadrants of Dunn’s Sensory Processing Framework, registration, seeking, sensitivity and avoiding, based on the child’s neurological threshold to sensory input and their method of self-regulation (Dunn, 1999). The SP was normed for typically developing children aged 3–14 years and 11 months (*n* = 1, 037) and has average to strong internal consistency (Cronbach's *α* = 0.47–0.91 across scales).

Raw score totals can be calculated for each sensory subscale and each quadrant. Dunn (1999) provides a Normal Curve and Classification System based on responses from a normative sample of children without disabilities (*n* = 1037). Based on a bell curved normal distribution, the raw score total for each scale can be classified as “definitely lower” (lower 2%), “probably lower” (between 1 and 2 *SD* below the mean, accounting for 14% of the normative sample), “typical” (± 1 *SD* from the mean and accounting for 68% of the normative sample), “probably higher” (between 1 and 2 *SD* above the mean), and “definitely higher” (upper 2%).

The Auditory, Visual, Vestibular, Touch and Oral subscales, as well as the subscales for each of the four quadrants of Dunn’s Sensory Processing Framework were utilised. The Multisensory subscale was not utilised in this study to allow meaningful comparisons to the findings of T2, which utilises the Sensory Profile 2 (SP2) and has the Multisensory subscale removed (Dunn, 2014).

## Statistical Analysis

SPSS version 26 was used to conduct the analyses and descriptive statistics for all key variables were generated. A hierarchical agglomerative cluster analysis using Ward’s method and the Square Euclidean distance measure was conducted to identify subgroups of people with similar sensory features (Hair, 2009). Total raw scores from each of the subscales were converted to standardised scores when entered into the analysis. The agglomeration coefficients and dendrograms were inspected to determine the number of clusters. The stability of the hierarchical Ward’s cluster solution for the respective samples was examined using a k-means cluster analysis with the number of clusters specified in advance based on the hierarchical cluster analysis solutions. Independent Samples T-Tests and Mann–Whitney U tests were used to determine the differences between each cluster due to normal and non-normal distributions on different scales.

## Results

### Overall Group Sensory Profile

Table 1 provides an overview of the scores for each sensory subscale for the entire group. Nearly all of the sample experienced impairments classified as “definitely higher” in the auditory sensory system (89.5%), whereas only a minority experienced “definitely higher” impairments within the visual sensory system (39%). The majority of the sample also experienced “definitely higher” impairments within the four quadrants of Dunn’s Sensory Processing Framework, registration (86.84%), sensitivity (86.86%) and avoidance (84.21%), whereas a smaller majority experienced “definitely higher” seeking behaviours (68.42%). The mean raw scores for the visual and touch subscales were in the “probable” impairment range, and the remaining subscales were in the “definitely higher” impairment range. All variables, apart from the ‘age’ and Auditory subscale, were normally distributed and had a skew and kurtosis figure ranging between − 2 and + 2.

**Table 1:** Overview of scores per sensory subscale for the entire WS group

Subscale	Mean (SD)	Sample range	Definitely impaired classification	Percentage of sample classified as definitely impaired
Auditory	20.5 (4.64)	8–32	8–25	89.5
Visual	27.42 (5.3)	18–35	9–26	39
Vestibular	41.16 (7.1)	26–53	11–44	60.5
Touch	66.18 (10.8)	53–85	18–64	50
Oral	38.92 (9.4)	21–54	12–39	55.26
Registration	44.26 (10.23)	26–65	15–58	86.84
Seeking	87.76 (12.22)	65–108	26–91	68.42
Sensitivity	60.92 (11.7)	32–82	20–72	86.84
Avoidance	87.5 (13.31)	59–108	29–102	84.21

### Cluster Analysis

**Fig. 1:**
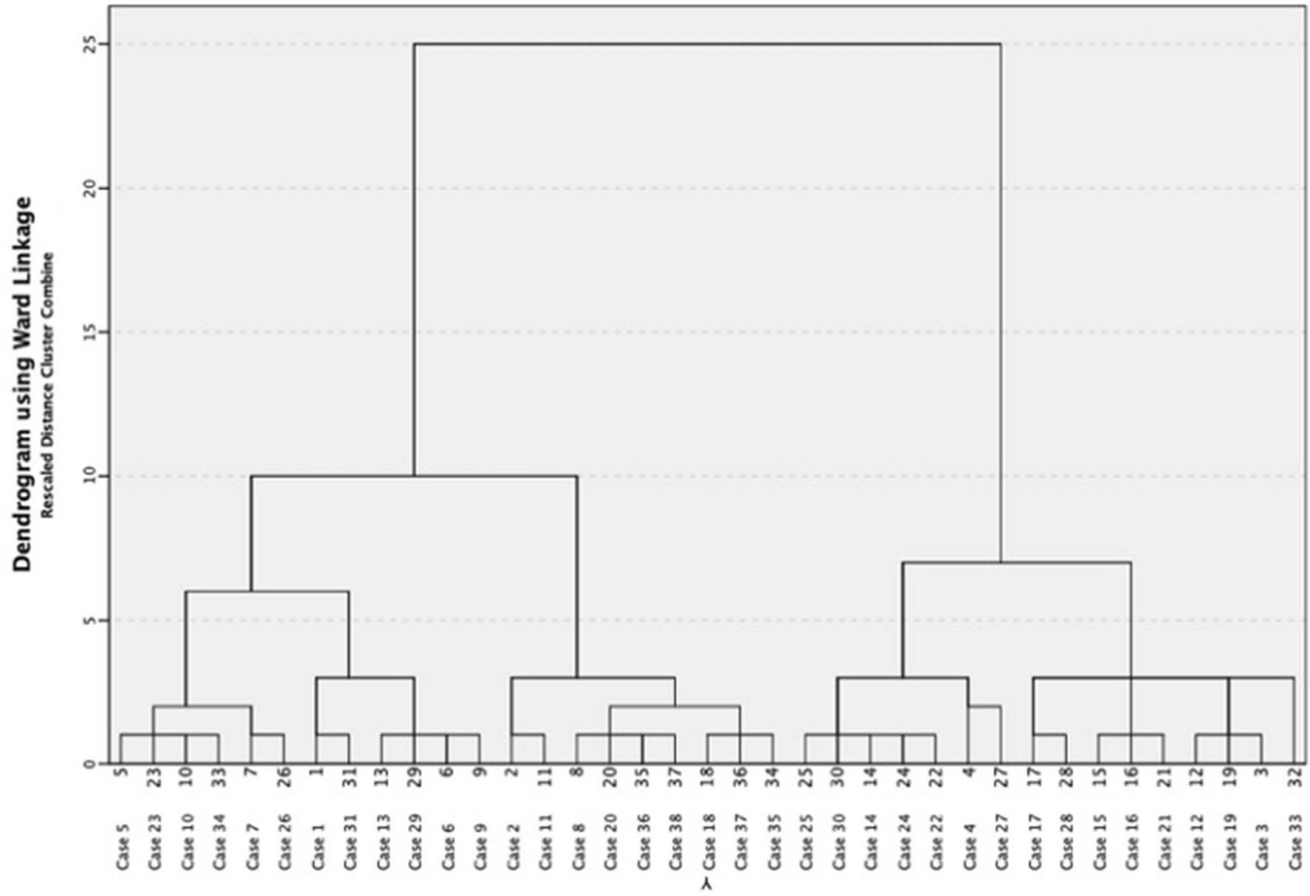
Dendrogram utilising z-scores across each sensory system using Hierarchical cluster analysis with Wards Method and Square Euclidian distance

Figure 1 shows the dendrogram generated by the cluster analysis which suggested a two-cluster solution. These two clusters included a similar number of participants (see Table 2). There was no significant difference for age between the two groups, even though those in the high impairment cluster were older by 23 months on average (*p* > 0.05).

**Table 2:** Descriptive statistics for each cluster and differences between the clusters

	Cluster 1 Mean (SD)	High impairment group	Cluster 2 Mean (SD)	Low impairment group	*t*	*p*	*d*
N	18		19				
Age	83.83 (40.29)		61.05 (17.48)		128.50**	.196	0.73
Auditory	18.33 (4.42)	Definite	22.63 (4.03)	Definite	67.50**	.002*	1.02
Visual	26.06 (5.64)	Definite	29.05 (4.56)	Probable	− 1.781	.084	0.58
Vestibular	38.33 (7.55)	Definite	44.42 (5.09)	Probable	− 2.861	.008	0.94
Touch	59.56 (8.05)	Definite	73.11 (2.00)	Typical	− 4.898	.001*	2.31
Oral	33.06 (6.16)	Definite	44.37 (9.00)	Typical	− 4.437	.001*	1.45
Registration	38.50 (7.97)	Definite	50.32 (8.72)	Definite	− 4.482	.001*	1.41
Seeking	83.67 (11.12)	Definite	91.47 (12.58)	Probable	− 1.995	.054	0.65
Sensitivity	51.94 (8.46)	Definite	69.89 (6.61)	Definite	− 7.214	.001*	2.36
Avoiding	78.94 (8.67)	Definite	97.11 (8.79)	Definite	− 6.323	.001*	2.08
Total		9/9		4/9			

When comparing cluster mean scores, a lower mean indicates greater impairment. ** Indicates Mann–Whitney U test

^*^Indicates significant results. Alpha level *p* < .006 due to Bonferroni correction

Cluster 1 showed uniformly elevated scores indicating “definitely higher” impairments across all subscales. Cluster 2 had less severe impairments, scoring in the “typical” range for the Touch and Oral subscales and “probable” impairments for the Visual, Vestibular and Seeking subscales. The remaining subscales for Cluster 2 were classified with “definitely higher” impairments (see Table 2). The two-cluster solution was validated by the k-means cluster analysis and showed good agreement as 96% of the participants kept their cluster membership in the k-means cluster solution.

The differences between the clusters were further examined with Independent Samples T-Tests. Mann–Whitney U tests were used to assess the differences between the Clusters scores on the Auditory subscale and mean age due to non-normal distributions (Table 2). Whilst using Bonferroni correction, the mean raw scores across all subscales, apart from the Visual, Vestibular and Seeking subscales, were significantly different between the two clusters (*p* > 0.006).

## Discussion

Experiment 1 examined individual differences in sensory processing difficulties using a cross-sectional sample of children with WS similar to John and Mervis (2010). The current study provides further insight into the impairments experienced by children with WS, given that the long form SP provides scales to individually measure registration and sensory seeking, in addition to sensitivity and avoidance.

Overall, the majority of the children experienced “definitely higher” impairments in the Auditory, Registration, Sensitivity, and Avoidance subscales. The finding that there are high rates of sensitivity and avoidance sensory processing difficulties is novel. It is likely that a high degree of sensory sensitivity may contribute to greater rates of sensory avoidance, due to the discomfort associated with greater sensitivities (Crane et al., 2009). Only a minority of the participants experienced “definitely higher” impairments in the Visual subscale (39%). While past research has widely documented the prevalence of auditory sensory processing impairments within WS (Gallo et al., 2008; Gothelf et al., 2006; Honjo et al., 2015; Klein et al., 1990; Udwin, 2010), little research has examined the development of general sensory processing (Glod et al., 2019). Previous research using the SSP had identified sensory registration, seeking and hypotonia to be a source of difficulty for children with WS (John & Mervis, 2010). Yet, the current study also distinguishes that children with WS experience impairments across each sensory processing quadrant of Dunn’s (1997) framework, thereby contributing to impairments across each of the five sensory modalities. The current study utilised the SP long form, which provides a more detailed analysis of a child’s sensory processing characteristics in comparison to the SSP, which may explain the greater prevalence of certain sensory impairments within our sample.

The results of the cluster analysis are in line with the previous finding that there are two homogenous groups of sensory processing ability within children with WS; *high* and *low impairment* (John & Mervis, 2010). The present study extends this research through identifying the sensory differences between the two clusters. The *high impairment* group displayed a severely impaired sensory profile, with elevated scores in the “definitely higher” difference range across each subscale. Whereas the *low impairment* group displayed a complex and mosaic sensory profile, displaying “definitely higher” impairments in the Auditory, Registration, Sensitivity and Avoidance scales. The two clusters significantly differed on all but the Visual, Vestibular and seeking subscales. The effect sizes were especially large on the touch and oral sensitivity scales (*d* = 2.30), further indicating that the *high impairment* group experiences significantly greater impairments. The two clusters did not differ in age, which suggests that age differences do not explain cluster membership.

These results provide evidence that the childhood WS phenotype is characterised by sensory impairments, particularly with regards to the auditory sensory system and the registration, sensitivity and avoidance sensory quadrants. The results also suggest that children with WS will either experience *high* or *low sensory impairments*, and that those in the *low impairment* cluster will have considerably greater sensory processing ability than those is the higher sensory impairment group, especially in touch and oral sensory processing while still experiencing impairments in all other areas. The results have important implications for practice, given that sensory processing is an essential component of adaptive functioning. Understanding the individual variability in sensory processing in children with WS allow to tailor interventions and support, thereby enhancing adaptive functioning.

Notwithstanding, as the data was extracted from a longitudinal dataset, additional participant details were not available. Understanding the effect of co-morbidities, occupational therapies, education and parenting is important given that these can impact upon sensory processing development (Allen & Casey, 2017; Ben-Sasson et al., 2013; Kern et al., 2007; Pfeiffer et al., 2011). In addition, it is not clear whether these two subgroups are stable over development.

## Experiment 2

As previous studies in sensory processing in typically developing populations and autistic individuals have shown that sensory processing difficulties decrease with age (Little et al., 2017), the trajectory of sensory processing development in WS and the stability of the clusters established in Experiment 1, were examined using a longitudinal design.

## Participants

The thirty-seven parents from Experiment 1 were contacted for this follow-up study. Contact details were no longer accurate for nine of the parents and 15 did not respond, possibly due to the increased pressures on families related to COVID-19, leaving 16 to complete the follow-up survey measures (four females). The average age of the longitudinal participants at Timepoint 1 (T1) was 6.6 years old (SD = 3.4). At Timepoint 2 (T2), the average age of the participants was 8.8 years old (SD = 4.1). The time between T1 and T2 varied between 1.1 and 4.4 years (SD = 1.2 years). This project received ethical approval from the Faculty Ethics Committee (REC1323). As we did not have any background data for participants in Experiment 1, it is not possible to comment on the representativeness of this subset of participants.

## Materials

The SP2 (Dunn, 2014) was used and is a revised and updated version of the SP questionnaire. The main changes consist of reversed scoring and a slight reduced number of items. Parents reported on 86-items which measure children’s responses to everyday events in six sensory modalities (i.e. auditory, visual, touch, movement, body position and oral) and three behavioural modalities (i.e. conduct, social emotional and attentional). The SP2 also provides scores for each of the four quadrants of Dunn’s Sensory Processing Framework (i.e. registration, seeking, sensitivity and avoidance) just like SP1. The Likert scoring of the SP2 represents ‘0 = Not Applicable’, ‘1 = Almost Never’ to ‘5 = Almost Always’. The SP2 was normed for typically developing children aged 3–14 years and 11 months (*n* = 1791) and demonstrates strong internal consistency (Cronbach's *α* = 0.88–0.92 across scales) (Dunn, 2014). Raw score totals can be calculated for each sensory subscale and each quadrant. Dunn (2014) provides a Normal Curve and Classification System based on responses from a normative sample of children without disabilities (*n* = 1791). Reversed scoring of SP2 scores was done and then average scores per modality were calculated for SP1 and SP2 modalities separately to allow direct comparisons.

The SP2 was completed by the same caregiver as for Experiment 1.

## Statistical Analysis

SPSS version 26 was used to conduct the analyses and descriptive statistics for all key variables were generated. To assess overall group sensory processing stability, Fishers Exact Tests were conducted to assess whether classification at T1 was associated with classification at T2 (i.e. shift from “definitely higher” impairment to “typical” processing and vice versa). In addition, Wilcoxon-Signed Ranks test was used to test the difference between total number of scales classified with “definitely higher” impairments at T1 compared to T2. Participants average scale scores for each subscale at T1 and T2 was extracted and Independent Samples T-Tests and Mann–Whitney U tests were conducted to assess the group differences in average score from T1 and T2. As each participant differed on time elapsed between T1 and T2, an ANCOVA controlling for follow-up duration was conducted.

To explore individual sensory profile stability from T1 and T2, the total *N* of sensory systems impaired at both timepoints was extracted and visually compared. To assess the stability of the clusters from T1, Independent Samples T-Tests with Cluster Membership at T1 compared the T2 average scale scores for each subscale between the two clusters to assess whether the clusters continued to significantly differ on each subscale at T2.

## Results

### Overall Group Characteristics at T1 and T2

Overall group sensory processing characteristics are presented in Table 3. A Wilcoxon signed-rank test found there was a significant difference in the total number of sensory systems impaired for each individual at T1 (median = 6.5) to T2 (median = 2.00) (Z =  − 2.661, *p* = 0.008). Despite the decrease in the total number of participants classified with “definitely impairments at T2, Fishers Exact Tests’ found no significant association between the number of subscales classified as impaired at T2 in comparison to T1 (*p* > 0.05). This shows that although the number of participants identified to show impaired sensory processing across all of the subscales decreased, the participants still show impaired sensory processing across a wide number of subscales.

**Table 3:** Overall group sensory processing characteristics

	T1 Mean (SD)	*N* classified as ‘definitely different’	T2 Mean (SD)	*N* classified as ‘definitely different’
N	16		16	
Age	6.6 (SD, 3.4)		8.8 (SD, 4.1)	
Auditory	2.64 (.75)	11 (68.75%)	2.61 (.87)	6 (37.5%)
Visual	3.25 (.47)	4 (25%)	3.69 (.75)	3 (18.75%)
Vestibular	3.91 (.61)	8 (50%)	3.44 (.56)	5 (31.25%)
Touch	3.65 (.71)	9 (56.25%)	3.59 (.72)	5 (31.25%)
Oral	3.30 (.88)	9 (50%)	3.26 (.78)	7 (43.75%)
Registration	2.90 (.69)	14 (87.5%)	3.04 (.72)	12 (75%)
Seeking	3.37 (.56)	10 (62.5%)	3.56 (.56)	3 (18.75)
Sensitivity	3.15 (.47)	13 (81.25%)	3.13 (.60)	7 (43.75%)
Avoiding	3.23 (.34)	14 (87.5%)	3.07 (.56)	8 (50%)

### T1 and T2 Sensory Processing Scale Average

To assess the difference in sensory processing ability at T1 and T2, repeated measures t-tests were conducted. There was a significant increase from the Visual T1 (3.25, SD = 0.47) to the Visual T2 (3.69, SD = 0.75) average scale score; *t* (15) = -2.711, *p* = 0.016, *d* = 0.92, and a significant decrease from the Vestibular T1 (3.91, SD = 0.61) to the Vestibular T2 average scale score (3.44, SD = 0.56); *t* (15) = 2.710, *p* = 0.016, *d* = 0.92*.* The differences between the remaining subscales at T1 and T2 were non-significant, when correcting for multiple comparisons (*p* > 0.006).

As the time between T1 and T2 was different for each participant, a Repeated Measures ANCOVA controlling for follow-up duration was conducted. All average scale variables were normally distributed upon inspection of Shapiro Wilk, Histograms and QQ Plots. The Registration Average Scale Score and the Touch T2 Average Scale Score had outliers identified through datapoints outside of the whiskers of a boxplot. The outliers remained in the dataset to reflect the variation in SP ability within WS, and therefore the assumption of no outliers was violated. In addition, the DVs did not have a linear relationship with the covariate (follow-up duration) and the assumption of sphericity was violated. Therefore, the results must be interpreted with some caution. The results found that there was no significant effect for follow-up duration upon the difference between scores at T1 and T2 (Wilks *p* > 0.005), suggesting length of follow-up duration does not influence the results found with regards to Vestibular and Visual subscales in the study.

### Individual Sensory Profile at T1 and T2

To assess the stability of individual sensory profiles at T1 and T2, the total number of sensory systems impaired (minimum zero, maximum nine) at T1 was compared to T2 for each participant (see Table 4). Participants can be categorised into three different sensory development groups based on the total number of systems that were impaired at T1, and whether this figure remained stable or changed at T2 (see below).

**Table 4:** Comparison of sensory profile at T1 and T2, *n* of sensory systems classified as “definitely higher**”**

Group	Participant	Cluster at T1	T1		T2		Follow-up duration (years;months)
			Age (years;months)	*N* systems definitely impaired	Age (years;months)	*N* systems definitely impaired	
1	1	2	03;09	1	05;11	0	02;02
1	2	2	04;04	1	08;07	2	04;03
1	3	2	04;03	3	08;06	3	04;03
1	12	2	03;08	4	07;10	2	04;02
2	4	1	11;04	7	15;05	2	04;01
2	5	1	10;11	9	14;05	0	03;06
2	6	1	14;03	8	17;09	2	03;06
2	9	2	04;06	6	07;07	2	03;01
2	10	2	04;06	7	07;09	1	03;03
2	13	2	04;07	5	07;02	2	02;07
3	7	1	04;02	9	05;08	7	01;06
3	8	1	05;01	6	06;05	5	01;04
3	11	1	07;06	7	08;07	8	01;01
3	14	1	10;00	6	13;00	7	03;00
3	15	2	03;07	7	05;02	7	01;07
3	16	1	08;00	7	09;01	6	01;01

Group 1 is characterised by low impairments at T1 and at T2 (mean follow-up duration 2.8 SD = 1.49). Group 2 has high impairments at T1 and low at T2 (mean follow-up duration 2;08, SD = 1.16). Group 3 experiences high impairments at T1 and at T2 (mean follow-up duration 2;03, SD = 1;08)

The first group (low- low, *n* = 4) consists of individuals with a maximum of four systems classified with “definitely higher” impairments at both T1 (mean age: 4;04, SD = 0.78) and T2 (mean age: 6;08, SD = 0.74). The second group (high-low, *n* = 6) consists of individuals with a maximum of nine systems classified with “definitely higher” impairments at T1 (mean age: 6;06, SD = 3.4) and a maximum of two “definitely higher” impairments at T2 (mean age: 9;07, SD = 3.8). The final group (high-high, *n* = 4) consists of individuals with high impairments at T1 with a maximum of nine classified as “definitely higher” (mean age 7;02, SD = 4.3) and at T2, with a maximum of eight classified as “definitely higher”, (mean age 9;04, SD = 4;08). A One-Way ANOVA found there were no significant differences between the groups follow-up duration or age at T1 and T2 (*p* > 0.05).

### Stability of Sensory Clusters

Table 5 provides the descriptive characteristics of the clusters at both T1 and T2. As can be seen from the table, the mean scale average score for eight of the nine subscales were classified as ‘definitely’ different at both timepoints for Cluster 1. Similarly, Cluster 2 had five of the nine subscales at both timepoints. However, the total number of participants classified with “definitely higher” impairments appeared to decrease across both clusters at T2. Therefore, to assess the stability of the clusters found in Experiment 1, Independent Groups t-tests based on Cluster Membership at T1 compared the T2 scores for each subscale between the two clusters to assess whether the clusters continued to significantly differ on each subscale at T2.

**Table 5:** Cluster scores: mean (M) and standard deviation (SD), classifications (D = definite, P = probable, T = typical) and total *n* defined as definite across T1 and T2

	T1						T2					
	Cluster 1			Cluster 2			Cluster 1			Cluster 2		
	M (SD)	Classification	N (%)	M (SD)	Classification	N (%)	M (SD)	Classification	N (%)	M (SD)	Classification	N (%)
*N* = *16*	8			8			8			8		
Age		8.3 (3.82)		4.6 (1.3)								
Auditory	2.32 (2.32)	D	7 (44%)	2.96 (2.96)	D	4 (25%)	2.30 (.86)	D	4 (25%)	2.92 (.82)	D	2 (12.5%)
Visual	3.19 (.46)	P	2 (12.5%)	3.31 (.49)	P	3 (19%)	3.41(.81)	P	2 (12.5%)	3.98 (.60)	T	1 (10%)
Vestibular	3.76 (.68)	D	5 (31%)	4.06 (.53)	D	3 (19%)	3.32 (.67)	D	4 (25%)	3.56 (.44)	D	1(10%)
Touch	3.12 (.36)	D	7 (44%)	4.17 (.57)	T	2 (12.5%)	3.41(.86)	D	3(19%)	3.75 (.56)	P	2 (12.5%)
Oral	2.75 (.69)	D	7 (44%)	3.84 (.71)	P	2 (12.5%)	3.06 (.89)	D	5 (31%)	3.46 (.66)	P	2 (12.5%)
Registration	2.49 (.41)	D	8 (50%)	3.32 (.68)	D	6 (38%)	2.75 (.71)	D	7 (44%)	3.33 (.64)	D	5 (31%)
Seeking	3.08 (.44)	D	7 (44%)	3.66 (.68)	P	3 (19%)	3.51(.54)	D	1(10%)	3.60 (.60)	P	2 (12.5%)
Sensitivity	2.78 (.25)	D	8 (50%)	3.52 (.33)	D	5 (31%)	2.87 (.73)	D	5 (31%)	3.39 (.30)	D	2 (12.5%)
Avoiding	2.98 (.23)	D	8 (50%)	3.49 (.23)	D	6 (38%)	2.86 (.69)	D	6 (38%)	3.29 (.32)	D	2 (13%)
Total		8/9			5/9			8/9			5/9	

Despite including only half of the participants, these results were similar to that of the findings illustrated in Table 2 in Experiment 1. With the subsample whilst correcting for multiple comparisons, the two clusters scores at T1 differed significantly on the Touch; *t* (14) =  − 4.397, *p* = 0.001, *d* = 1.84, Oral; *t* (14) =  − 3.107, *p* = 0.008, *d* = 1.54, Sensitivity; *t* (14) =  − 4.929, *p* = 0.001, *d* = 2.42, and Avoiding; *t* (14) =  − 4.381, *p* = 0.001 *d* = 2.21, subscales, whereas the differences between the Auditory, Vestibular, Registration and Seeking subscales were no longer significant (*p* > 0.006). At T2, there were no significant differences between the clusters (all *p*’s > 0.006).

## Discussion

Experiment 2 is the first study to examine whether sensory processing difficulties decrease or remain stable across development in children and adolescents with WS using a longitudinal design. Across a one to four-year follow-up period, it was found that the total number of systems classified as definitely impaired at T1 significantly decreased at T2. However, further analyses suggest a nuanced picture. The total number of participants experiencing definite impairments in the registration scale remained relatively stable from T1 to T2, suggesting that impaired sensory registration is a stable phenotype for children with WS. In line with previous findings, the total *n* experiencing auditory impairments decreased substantially from T1 to T2, suggesting auditory impairment severity may be overcome with age in some children (Gallo et al., 2008; Gothelf et al., 2006; Honjo et al., 2015; Klein et al., 1990; Udwin, 2010).

When comparing the group scores at T1 and T2 for each subscale, there was a significant increase from the visual T1 to T2 average score, indicating a decrease in level of impairment for the group. In addition, there was a significant decrease from the vestibular T1 to T2 average modality score, indicating an increase in vestibular impairments. These results suggest that these individual sensory modalities follow different developmental trajectories, rather than each sensory system developing uniformly (Ben-Sasson et al., 2009; Little et al., 2017). Length of follow-up duration did not have a significant effect on these results. It is possible a larger sample with a wider age range may have found greater differences between T1 and T2 sensory processing, given that executive functioning may increase as a child ages, subsequently influencing sensory processing ability (Costanzo et al., 2013; Rueda & Rothbart, 2009). Furthermore, as a child shifts from middle to late childhood, there may be a change in the child’s environment such as a change in schooling or available interventions which may also influence sensory development trajectories (Allen & Casey, 2017; Ben-Sasson et al., 2013; Kern et al., 2007).

Three distinct groups of sensory processing developmental trajectories were identified by comparing how many sensory processing systems changed from T1 to T2 for each participant. The low-low group had low impairments at both T1 and T2. This shows that there are some children with WS that have few sensory processing difficulties across development. It is possible the children in this subgroup have greater executive functioning overall and subsequent capacity to process sensory information at both T1 and T2 (Miezah et al., 2020). The high-low group had high impairments at T1 and low impairments at T2. It is possible the participants in this group had an increase in cortical maturation and executive functioning ability, allowing greater processing capacity (Porter & Dodd, 2011) or that they had some intervention (Case-Smith et al., 2014) or a change in their environment. The high-high group had high impairments at both T1 and T2 and it is possible these participants may have poorer overall functioning due to impaired executive functioning and subsequent sensory processing (Carvill, 2008) or that they had additional co-morbidities that impact on their sensory processing difficulties (Dellapiazza et al., 2020). However further studies are needed to assess these possibilities. Yet, the groups did not differ significantly for age and there was no effect for the length of follow-up, suggesting these mechanisms do not influence the development trajectories.

The three different developmental pathways may explain why the two sensory clusters identified at T1 no longer significantly differed on any of the subscales at T2. For instance, within the high-low group, six participants experienced high sensory impairments at T1, and their total N of definite impairments decreased at T2. It is possible that the change of these participants sensory processing scores significantly influenced the results of the t-tests between the clusters. In addition, the overall number of participants was lower which means there was less power to identify significant differences. However, this is unlikely as the cluster differences for T1 were replicated in the sub-sample that was longitudinally followed-up. Alternatively, it is possible those who responded to T2 were a specific sub-group of children. However, this is less likely given that participants from both clusters responded. Instead, the overall results imply that sensory impairments for the majority of children with WS do decrease during childhood. Notwithstanding, there are participants who continued to experience high impairments at T2 which suggests a minority of individuals with high sensory impairments will continue to experience high impairments throughout childhood. Future studies may wish to examine how these individual differences relate to participants’ overall intellectual abilities and adaptive functioning.

## General Discussion

The two experiments in the current study examined the development of sensory processing difficulties in WS related to the sensory processing factors, and the individual variability within this group using both a cross-sectional as well as for the first time a longitudinal design. With regards to development over time, the two studies showed that overall there was a decrease in sensory processing difficulties. This finding is different from children with autism for whom it has been found that profiles remained stable or sensory difficulties increased in a small sample of participants (Dwyer et al., 2020; Perez Repetto et al., 2017). In addition, there were differences between the factors: whilst visual processing difficulties decreased, vestibular processing difficulties increased. Moreover, for the registration scale the decrease was minimal which suggests that impaired sensory registration difficulties may be stable throughout childhood for individuals with WS. Still, at both times children were classified with “definitely higher” impairments on a number of sensory processing factors.

With regards to individual profiles, the cross-sectional study supported that there were two distinct groups of sensory processing difficulties within WS, *high* and *low impairment* (John & Mervis, 2010). The two cluster groups showed particular differences with regards to touch and oral processing at T1. However, the results showed that these two groups of sensory processing difficulties within WS are not stable over time, as there were no differences between the two clusters at T2. Instead, there were three different developmental trajectories when comparing individual sensory profiles at T1 and T2. Further research is required to examine the factors that may influence these different profiles process, with regards to the child’s executive functioning abilities, comorbidities or interventions and other environmental changes. However, the age of the child itself was not a main factor.

In terms of limitations, it could be argued that the number of participants in the current study is rather small. Yet, seeing the rarity of the disorder, more than half of the studies on WS usually include fewer than 16 participants (Martens et al., 2008). As such, the sample size of Experiment 1 is rather large (N = 37), accounting for approximately 17.8% of the children with WS in the UK within this age category.^1^ This number of participants was obtained through the use of the WisDOM database and this suggests that a collaborative approach is required to study development in WS to allow data pooling from across different research centres and labs and increase the rigour of the research in terms of participant numbers.

For Experiment 2, a large number of the contact details for participants who took part in Experiment 1 were either no longer valid or could not be accessed. This is not uncommon in longitudinal studies, especially after new General Data Protection Regulation (GDPR) rules were agreed upon by the European Parliament and Council in April 2016 which meant that a lot of contact details could not be accessed anymore as parents had not given explicit permission that they could be contacted about future studies. Yet, as Experiment 2 included 16 participants, the current longitudinal study still included more participants compared to most cross-sectional studies in WS thus far (Martens et al., 2008). Furthermore, given the rarity of WS, the rate of attrition in the present study may also be comparable to that of other published research.

Although future research with longer follow-up times and older participants with WS, as well as possibly more participants, is needed, together the current results provide further evidence that sensory impairments are highly prevalent for children with WS, particularly impaired sensory registration, but that there seem to be individual differences with regards to touch and oral sensitivity. In addition, sensory processing difficulties change over time, especially visual and vestibular processing ones. These findings are of clinical importance as they show that all children with WS should be assessed for sensory processing difficulties, that these assessments should be reviewed to see how the processing difficulties change with age, and that interventions are required to ensure children with WS can manage their environments adaptively.

## Footnotes

1 Using the average of 1 in 18,000 live births (Martens et al., 2008) and the number of typically developing children in UK in this category (Clark, 2020) compared to number of participants included.
